# The Role of Syndecan-1 in Cellular Signaling and its Effects on Heparan Sulfate Biosynthesis in Mesenchymal Tumors

**DOI:** 10.3389/fonc.2013.00310

**Published:** 2013-12-19

**Authors:** Tünde Szatmári, Katalin Dobra

**Affiliations:** ^1^Department of Laboratory Medicine, Karolinska Institutet, Karolinska University Hospital, Stockholm, Sweden

**Keywords:** syndecan-1, heparan sulfate, signaling, cancer, mesenchymal tumor

## Abstract

Proteoglycans (PGs) and in particular the syndecans are involved in the differentiation process across the epithelial-mesenchymal axis, principally through their ability to bind growth factors and modulate their downstream signaling. Malignant tumors have individual proteoglycan profiles, which are closely associated with their differentiation and biological behavior, mesenchymal tumors showing a different profile from that of epithelial tumors. Syndecan-1 is the main syndecan of epithelial malignancies, whereas in sarcomas its expression level is generally low, in accordance with their mesenchymal phenotype and highly malignant behavior. This proteoglycan is often overexpressed in adenocarcinoma cells, whereas mesothelioma and fibrosarcoma cells express syndecan-2 and syndecan-4 more abundantly. Increased expression of syndecan-1 in mesenchymal tumors changes the tumor cell morphology to an epithelioid direction whereas downregulation results in a change in shape from polygonal to spindle-like morphology. Although syndecan-1 plays major roles on the cell-surface, there are also intracellular functions, which are not very well studied. On the functional level, syndecan-1 affects mesenchymal tumor cell proliferation, adhesion, migration and motility, and the effect varies with the different domains of the core protein. Syndecan-1 may exert stimulatory or inhibitory effects, depending on the concentration of various mitogens, enzymes, and signaling molecules, the ratio between the shed and membrane-associated syndecan-1 and histological grade of the tumour. Growth factor signaling seems to be delicately controlled by regulatory loops involving the syndecan expression levels and their sulfation patterns. Overexpression of syndecan-1 modulates the biosynthesis and sulfation of heparan sulfate and it also affects the expression of other PGs. On transcriptomic level, syndecan-1 modulation results in profound effects on genes involved in regulation of cell growth

## Syndecan Structure

**Figure 1:**
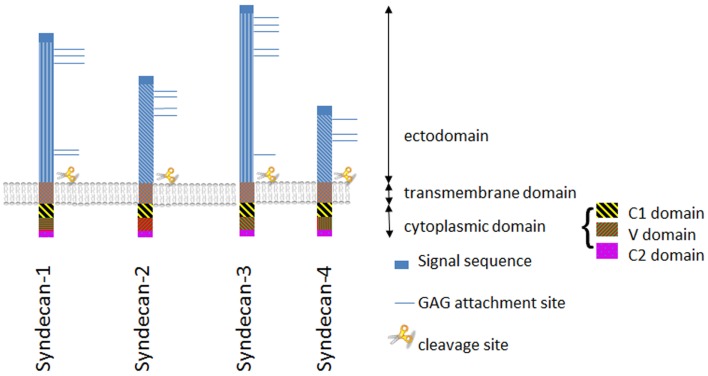
**The syndecan family**. Schematic illustration of structurally related syndecan genes, showing the two subfamilies of syndecans: syndecan-1 and -3, and syndecans -2 and -4, respectively. The extracellular domain is highly variable with the exception of the GAG attachment sites and the proteolytic cleavage site near the plasma membrane. In contrast the endo- and transmembrane domains are well preserved.

**Figure 2:**
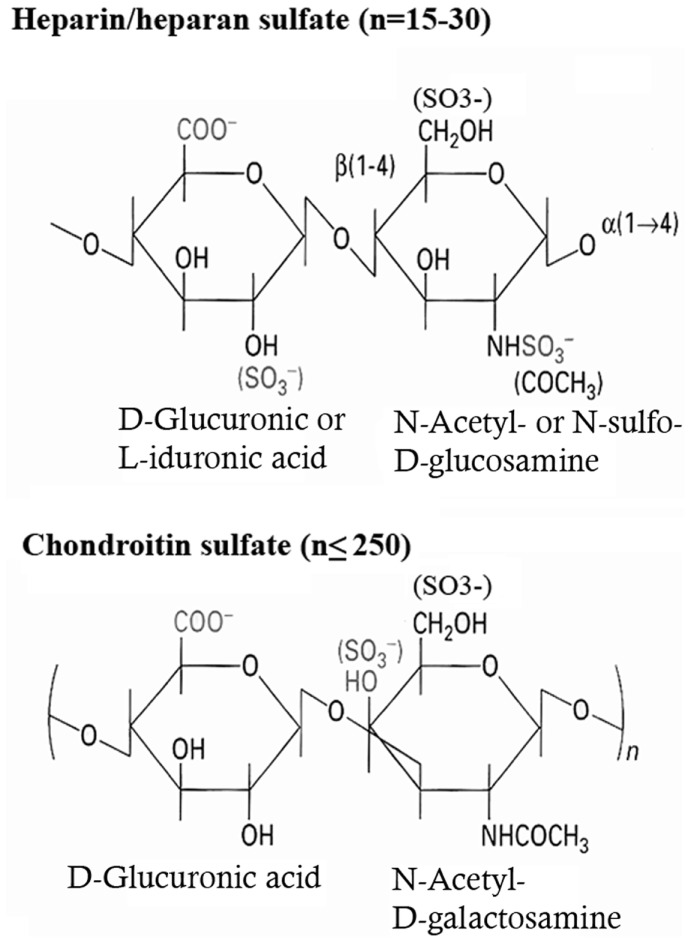
**Biochemical structure of the repeating disaccharide units of heparan sulfate and chondroitin-sulfate**.

Syndecans are transmembrane proteoglycans (PGs) composed of a core protein to which growth factor binding glycosaminoglycan (GAG) side chains are attached. The syndecan family consists of four members. Syndecan-1 is the major syndecan of epithelial cells (1), syndecan-2 is present mainly on cells of mesenchymal origin (2), syndecan-3 is primarily found in neuronal tissue and cartilage (3, 4), and syndecan-4 is ubiquitously expressed (5, 6). The protein cores of syndecans consist of a highly conserved C-terminal cytoplasmic domain, a single-pass transmembrane domain and a large N-terminal extracellular domain (7, 8) (Figure 1). The ectodomain carries up to five GAG chains, and syndecan-1 from different tissues display different GAG types comprising heparan sulfate (HS) and chondroitin-sulfate (CS) of varying length and fine structure (9) (Figure 2).

Both HS and CS are attached to serine residues via the same linkage sequence (Xylose-Galactose-Galactose-Glucuronic Acid). Following the synthesis of this sequence, the first hexosamine derivative – *N*-acetyl-glucosamine (GlcNac) or *N*-acetyl-galactosamine (GalNac) – is added; this step being the decisive for the type of GAG subsequently formed. The basic GAG chain for both HS and CS then extends in the Golgi by further repetitive addition of glucuronic acid (GlcA) and a hexosamine derivative, which for HS is GlcNAc and for CS GalNAc. The resulting GAG is thus built up of repeating disaccharide units, consisting of an uronic acid and a hexosamine derivative (10).

Subsequently this basic structure is modified by a series of reactions (epimerization, deacetylation, sulfation), which occur in a tissue-specific manner. Particularly in HS, this generates a vast diversity of the fine structure and hence alters the capacity of this GAG to bind to other structures. These modification reactions thus represent one way to regulate the protein binding capacity of PGs.

### Shedding of syndecans from the cell surface

Syndecans are usually present on the cell surface (1, 7, 11), but they can also be released by the action of sheddases or accumulate in the cell nucleus (12); in the tumor stroma (13), and in body fluids (14–17). Shedding of the ectodomain is biologically important, converting the cell-bound syndecan to a soluble active ligand. Syndecan-1 shedding is regulated by matrix metalloproteinases (MMPs), including MMP-7, MMP-9, membrane-bound metalloproteinases (MT-MMP1), and a disintegrin and metalloproteases (ADAM10, ADAM17) (18–22). They act by proteolytical cleavage of the juxtamembrane site of the core protein. The mechanism of shedding is currently not completely understood (23), but recently a new mechanism where MMP-9 enhances syndecan-1 shedding via suppression of miR-494 was described (24). Accelerated shedding is mediated by MMPs (21), Rab-5 (25), growth factors (GFs) (26), heparanase, and HS (27, 28). It is also known that FGF-2 activates MMP-7 mediated shedding (29) and heparanase accelerate MMP-9 mediated shedding of syndecan-1 (27). Cell surface HSPGs can themselves participate in regulation of metalloproteinases, anchoring them to the cell surface via the GAG chains (23), and particularly for syndecan-1 it was demonstrated that the HS chains on the core protein suppresses shedding (28), giving an additional explanation on the above mentioned role of heparanase in this process.

The released ectodomain carries intact GAG chains, thus it has preserved ability to modulate growth factor responses and biological processes. Experimental studies have shown that membrane-bound and soluble forms of syndecan-1 have opposing effects on cancer cell functions (19, 30–33). High level of shed syndecan-1 is associated with infection, inflammation, and cancer. Recently it was found that chemotherapy can induce shedding of syndecan-1, particularly via ADAMs and this shed syndecan-1 being functionally active, leads to establishment of a more aggressive phenotype in case of relapse (34).

Soluble syndecan-1 binds pro-angiogenic factors like VEGF or FGF, activates them, creating a chemotactic gradient, and by this promotes endothelial cell’s invasion and angiogenesis. Besides, it activates the integrins αVβ3 and αVβ5 (35), which are also important for angiogenesis (36) and regulates the association of IGF1R to αVβ3 integrin, essential in endothelial cell migration (37). The pro-angiogenic effect of syndecan-1 was shown in myeloma (27), medulloblastoma (24), and in a variety of tumors of epithelial origin like endometrial cancer (38), breast cancer (39), and in stromal fibroblasts of breast cancer (32), but the effects of syndecan-1 in the angiogenesis of mesenchymal tumors is largely unknown.

Understanding of the importance of syndecan-1 shedding might help to resolve the seemingly contradictory expression levels documented in various malignancies.

### Sub-cellular localization of syndecan-1 in the cell nucleus

Syndecan-1 translocates to the cell nucleus of various tumor cells, including malignant mesothelioma, fibrosarcoma, neuroblastoma, breast- and lung adenocarcinoma, and multiple myeloma (12, 40–42). This translocation is tubulin dependent and the same transport route operates also for FGF-2 but not for the FGF Receptor. The minimal structural requirement for nuclear translocation is the RMKKK sequence at the cytoplasmic tail of syndecan-1 serving as a nuclear localization signal (NLS) (43). The nuclear translocation correlates to the differentiation and proliferation of certain tumor cells. One compound that seems to modulate the level of nuclear syndecan-1 in several tumor types is heparanase when simultaneously present in the nucleus (41, 44). High level of heparanase implies low levels of nuclear syndecan-1 and increased histon acetyl transferase (HAT) activity leading to enhanced transcription of VEGF and MMP-9, both known to drive an aggressive tumor phenotype (42). Furthermore, syndecan-1 restoration diminishes the nuclear HAT activity, providing a mechanistic link and establishing syndecan-1 as a powerful inhibitor of HAT driven gene transcription.

Accumulating evidences suggest that the localization of syndecan-1 might be crucial for its function and the nuclear translocation adds additional complexity which needs to be further addressed in the context of variously differentiated tumor components.

## Tissue- and Tumor Specific Expression of Syndecan-1

Each syndecan is expressed in highly regulated cell-, tissue-, and developmental stage specific manner (8, 45). The syndecan-profile of different tissues, hence of different tumor types, differs greatly between mesenchymal and epithelial tumors.

Similar to the normal epithelial cells, syndecan-1 is overexpressed in epithelial malignancies. In dedifferentiated tumor components and mesenchymal tumors its expression is, however, lower than in the parental tissue. Changes in syndecan-1 level can have remarkable consequences for tumor cell behavior. The expression of this PG can associate to disease stage, tumor differentiation grade, and prognosis of the tumor, though the extent and even the direction of the association varies from one tumor type to another (46).

The mechanisms by which syndecan-1 regulates tumor cell behavior are complex, and depend, at least partly, upon the interplay between tumors and the surrounding matrix. The expression of syndecan-1 and its role as a stimulatory or inhibitory factor probably depends upon the concentration of various mitogens, enzymes, and signaling molecules that are specific for each cancer type and histologic grade. By interacting with such factors, this PG modulates cancer cell proliferation, adhesion, migration, and angiogenesis.

## Syndecan-1 as Diagnostic and Prognostic Marker

Syndecan-1 is used as a standard diagnostic biomarker in multiple myeloma (47) and it is highly expressed in various human cancers (48) comprising pancreatic (49) and breast cancer (50). Hovewer, low cell surface syndecan-1 level is associated with a poor prognosis as demonstrated by immunohistochemistry in lung cancer (51), renal carcinomas (52), head and neck cancer (53), and in colorectal cancer (54, 55). In squamous cell carcinoma of the tonsil the level of syndecan-1 was found lower than in the benign keratoacanthoma and it correlated inversely with the proliferative index (56). In carcinoma of the uterine cervix expression of syndecan-1 is associated with histological differentiation grade but not with clinical outcome (57). Thus, syndecan-1 seems to have antithetic roles in different cancer types having inhibitory role on tumor formation and progression in many different epithelial malignancies but also promoting the growth of others (48, 58). The shed syndecan-1 can act opposing compared to cell-surface syndecan-1, since potentially it is able to sequester the GFs and other HS-binding soluble factors from the microenvironment of the tumor cell. Accordingly, the levels of shed syndecan-1 in serum correlate with a less favorable prognosis in lung cancer (16), lymphoma (59), myeloma (15), hepatocellular carcinoma (60), and glioma (61).

The tumor stroma has an important role in mediating tumor cell proliferation and invasiveness, leading to formation of metastases. In most tumor types the tumor stroma is abundant in matrix PGs, particularly versican, lumican, and fibromodulin, and this suggests an important role of stromal PGs in controlling tumor progression (58). The effects of shed syndecan-1 in the stroma is in majority of tumor types in contrast to those of cell-surface syndecan-1, the abundance of syndecan-1 in the tumor stroma being a negative prognostic factor (58) and it correlates to a more aggressive phenotype (33, 50, 62). Consequently, stromal syndecan-1 promotes breast epithelial cell proliferation (13); and in gastric cancer, ovarian cancer (63), and oral carcinoma (64) was associated with poor outcome (65). Similarly, in colorectal cancer immunoreactivity to syndecan-1 could be seen in both the tumor epithelium and stroma, whereas the normal colonic mucosa was negative for syndecan-1 (55). In basal cell carcinoma the opposite effect could be observed, where the stromal immunoreactivity of syndecan-1 inversely correlated to aggressiveness (66). Taken together, syndecan-1 seems to have an important role for epithelial-stromal interactions and a syndecan-1 dependent reciprocal feedback-loop has been proposed (67).

## Syndecan-1 in Mesenchymal Tumors

In mesenchymal cells the syndecan-1 level is usually low, but it is elevated transiently during embryonal morphogenesis (68–72) concomitant with a loss of syndecan-1 in the adjacent epithelium. Given this low syndecan-1 level in mesenchymal tumors, the expression, and function of syndecan-1 is far less studied than in carcinomas. The most extensively studied mesenchymal tumors addressing syndecan-1 expression are malignant mesothelioma and fibrosarcoma. Tumor cells forming this PG, however, have also been found in epithelioid components of biphasic sarcoma, thymoma, synovial sarcoma, leiomyosarcoma, gastrointestinal stromal tumors, and schwannomas (73, 74). Furthermore, a recent study revealed that in bone metastasis of soft tissue sarcoma syndecan-1 expression is elevated and it correlates to expression of several growth signaling molecules (75).

### Malignant mesothelioma

Cell-surface expression of syndecan-1 is relatively low in malignant mesothelioma compared to epithelial malignancies, however, its expression relates to epithelioid differentiation thus correlating to better prognosis (76), and it is reduced or lost in the sarcomatoid phenotype. Malignant mesothelioma cells also synthesize syndecan-2 and -4 and these syndecans, less often expressed in carcinomas, are especially abundant in the epithelioid phenotype (77). Thus syndecan-1 and syndecan-2 has been proposed as biomarkers to distinguish malignant mesothelioma from metastatic adenocarcinoma (78, 79).

Epithelial-mesenchymal transition is a characteristic feature of malignant mesothelioma (80) and *in vitro* model systems can be generated to mimic mesothelioma differentiation (81, 82). The mesothelium itself has a remarkable plasticity and a potential to generate other cell-types (83), whereas the mesothelioma has the ability to trans-differentiate across the epithelial-mesenchymal axis and this has prognostic significance (84, 85). This ability to switch from epithelial to mesenchymal phenotype involves a simultaneous downregulation of epithelial markers including syndecan-1 and E-cadherin (46, 86, 87).

The tumor microenvironment and growth factor gradients have a considerable effect on mesothelioma morphology and by modulating the serum composition of cell cultures the morphological changes of mesothelioma cells mimics various differentiation states *in vitro* (82). Molecular characterization reveals specific proteoglycan profiles and distinct molecular signatures for the epithelioid and sarcomatoid phenotypes, respectively (77, 88, 89). Experimentally induced overexpression of syndecan-1 in mesenchymal tumors changes the tumor cell morphology in an epithelioid direction (90), whereas downregulation results in a change in shape of cells from polygonal to spindle-like (77). At the same time, such overexpression inhibits tumor growth (90) and migration (43) of malignant mesothelioma cells simultaneously with enhanced cell adhesion.

### Fibrosarcoma

Fibrosarcomas are relatively rare malignant mesenchymal tumors, originating from fibroblasts, with an abundant extracellular matrix, rich in PGs. Though the amount of syndecan-1 is usually low in fibrosarcoma, some samples and cell lines can express also this PG (73, 90). Different studies show that this expression can modulate the proliferation, migration, and the malignant potential of tumor cells. Similar to carcinoma cells, however, the effects are cell-type dependent and seem to be governed by the spatio-temporal expression of syndecan-1. Variously differentiated tumor components behave differently: the proliferation and migration of a sarcomatoid fibrosarcoma cells is inhibited (43, 90) whereas in an epithelioid fibrosarcoma cell line is enhanced upon syndecan-1 overexpression (91, 92) in collaboration with syndecan-2 (92). In fibrosarcoma cells the location of syndecan-1 seems to be crucial. While cell-membrane-bound syndecan inhibited migration on collagen, the membrane type 1 metalloprotease (MT1-MMP) mediated shedding enhanced it (19).

## The Role of Syndecan-1 in Signaling

Syndecan-1 exerts mainly its functions via the HS chains, which ligate to a wide range of proteins, including heparin-binding GFs and their corresponding receptors, comprising FGFs, VEGF, Wnt, and HGF (27, 33, 93, 94). This ability to bind GFs is dependent on the steric orientation of the sulfate and carboxyl groups in the GAG chains. When simultaneously binding to both the growth factor and its receptor, HS stabilizes the complex, thus acting as a signaling co-receptor (11, 95). Most studies dealing with the effect of syndecans on signaling, have been performed in carcinomas or hematological malignancies (33, 96–98); thus the function of syndecan-1 is less studied in mesenchymal cells.

We have recently shown that overexpression of syndecan-1 in a malignant mesothelioma cell line influences a multitude of signaling pathways. These effects are not limited to cell-surface receptors but also influence their downstream effectors (99). The PDGF and FGF family members were downregulated, while their receptors were upregulated, whereas both the growth factor and its receptor were enhanced in EGF signaling. These changes in growth factor expression were accompanied by a deregulation of kinase cascade (ERK/MAPK, JNK, and p38/MAPK) and downstream transcription factors comprising MYC, FOS, JNK, and JUN which all were inhibited. On the other hand ETS-1 was upregulated due to syndecan-1 overexpression and inhibited when this PG was silenced. The direction or the magnitude of the effect seem to be cell-type specific, and does not allow direct extrapolation to other cells. Thus, ETS-1 was reported in colon carcinoma as inversely correlated to the level of syndecan-1 (100). Hence, growth factor signaling seems to be delicately controlled by the syndecan expression level, probably involving autoregulatory loops.

## Syndecan-1 Regulates the Expression of Enzymes Involved in Heparan Sulfate Biosynthesis and the Proteoglycan Profile

The HS chains are important in regulation of cancer cell behavior, different studies reported modified sulfation pattern of HS chains during cancer progression (101). Already 20 years ago it was assumed that the HS and PGs of a cell are subject of a coordinated regulation, and this regulation is critical for controlling cell behavior (102). Accumulating data support this, indicating a complex interplay between different proteins involved in the synthesis and turnover of heparan sulfate proteoglycans (HSPGs) (103, 104).

The original concept about HS biosynthesis highlights its specificity, proposing that it is a regulated, hierarchical process, comprising steps in a defined order, depending on each other. The enzymes *N*-deacetylase/*N*-sulfotransferase (NDST) replace the acetyl group of GlcNac with a sulfate group. As the substrate for NDSTs are the chains polymerized by exostosins (EXT), to which NDSTs add sulfate groups deriving from 3′-Phosphoadenosine-5′-phosphosulfate (PAPS), their activity depends on PAPS synthases and on EXTs as well. This N-sulfation is a key step for the consecutive 2-, 6-, and 3-O-sulfations, as the 2-*O*, 6-*O*, and 3-*O* sulfotransferases (2-OST, 6-OST, and 3-OST, respectively) add sulfate groups to the respective positions of disaccharide units in a strictly regulated order. This succession of events can explain why some of the HS chains are extensively modified, while others could remain totally unprocessed (10). The fact that the structure of HS chains correlates to the cell-type from where they are originated, rather than the core proteins which they bind, also points to a controlled expression of the HS biosynthetic enzymes. The mechanism behind synthesis of defined non-random HS sequences, so important for specific interactions, is still much of a mystery.

Proteoglycan and heparan sulfate biosynthesis are critical for development, morphogenesis, and organogenesis. Studies using different model organisms with one or more HS biosynthetical genes knocked out, show distinct severe developmental disorders and phenotypic deficiencies (105). The absence of any of these enzymes has serious implications in the morphogenesis and development of these organisms. Despite the tight regulation, there is a high degree of plasticity in the sulfation pattern, as a result of this flexible HS biosynthesis (106). Some observations does not support the model for HS biosynthesis where one enzyme creates the substrate for the next step, indicating that this order is not so strict, exemplified by the presence of 6-O-sulfation in HS lacking *N*-sulfate groups (107). These enzymes may interact as evidenced by studies in different model organisms (fruit fly, nematode, zebra fish, mouse) where knockdown of one of these enzymes is followed by direct and indirect effects, affecting the other enzymes from the HS biosynthetic machinery [for review see (108)]. Experiments with mice lacking NDST1 and/or NDST2 has shown that the HS N-sulfation is not limited by the total amount of active NDST enzymes (109); in mice deficient in C5 epimerase the HS N-sulfation is increased (110); and compensatory effects of 6-O-sulfation for 2-O sulfation were noticed (111, 112). Another study points out that 6-OST acts at the internal *N*/sulfoglcosamine and non-reducing terminal *N*-sulfoglucosamine but not *N*-acetylglucosamine *in vitro*, while *in vivo* all these residues are sulfated, indicating a coupled reaction of the enzymes (105).

Recently, it was hypothesized that instead of the total amount of an enzyme in one stage of the HS synthesis the critical step of the regulation may be the assembly of enzyme complexes of the cell (109). The idea was raised that the different biosynthetic enzymes form also physical complexes (10). Indeed, several studies have shown physical interactions of these enzymes: polymerases EXT1 and EXT2 (113, 114) as well as NDST1 and EXT2 (109, 115) form a complex and similarly, 2-OST and the epimerase co-localize in Golgi and interact physically (116), although this regulation is not always coordinated (117).

When trying to disclose the regulation of HS biosynthesis, we have to consider also the fact that in mammals almost all biosynthetic enzymes have more than one isoforms and the substrate specificities of different isoforms largely overlap. Moreover some of the genes encoding HS biosynthetic enzymes are regulated posttranslationally (10, 118), giving further complexity to the process.

Beside the above mentioned dynamic co-operativity of the HS biosynthetic enzymes in the Golgi, the interplay between the sulfatases (SULF-1 and SULF-2) and the synthesizing enzymes also play a role in the formation and function of the complex heparanome (108). The cell surface associated extracellular sulfatases remove 6-*O* sulfate groups from well-defined regions of the mature HS chains (119, 120). Loss of SULF-1 and/or SULF-2 results in different HS chain composition *in vivo*. In SULF-1 knockout cells, in addition to the increase in 6-*O* sulfated disaccharides, 2-O sulfation, and N-O sulfation decreased in small, but significant extent (121). Moreover, upon SULF-2 knockdown, SULF-1 is able to compensate its effect, while double knockouts showed synergistic co-operativity, resulting in a supraadditive effect and increased amount of 6-*O* disaccharides (108).

There are also evidences that HS and CS biosynthesis affects each other, possibly by sharing the same linkage regions; the absence of one allowing the other to substitute. Furthermore, they share the common PAPS pool for sulfation (117).

The regulation of these enzymes implies a “balanced hierarchy” between their activity and expression, finally resulting in a complex, cell-type specific sulfation pattern (108). Many pieces of this puzzle and the dynamic interplay still remain to be elucidated. The picture is complex, as the interactions are built up by both negative and positive feedback loops which can be depicted in a network (122), where each member reciprocally affects multiple actions of the other members of the network. Thus changes in the expression of one gene will affect the whole HS biosynthetic machinery of the cell.

A threefold overexpression of syndecan-1 in malignant mesothelioma largely influenced the whole transcriptome often with a much higher deregulation of individual genes than the syndecan-1 itself (99). Thus it seems that also other, post transcriptional or epigenetic mechanisms might contribute to these effects. One way of this regulation seems to involve the sulfation pattern of the HS chains. Syndecan-1 overexpression caused a significant alteration in the expression level of several enzymes involved in HS biosynthesis, metabolism, and turnover. In this setting EXT1 and NDST1 were downregulated along with deregulation of 2-*O*, 6-*O* sulfotransferases and the two PAPS synthases, responsible for the synthesis of the sulfate donor PAPS (99) (Table 1). Furthermore, SULF-1 was highly downregulated, though, the level of SULF-2 was not affected.

**Table 1:** **List of genes encoding proteoglycans and heparan sulfate biosynthetic enzymes affected by syndecan-1 overexpression in a mesothelioma cell line**.

Gene	Protein name	FC
**PROTEOGLYCANS**		
SDC2	Syndecan-2	−3.0
GPC3	Glypican-3	1.8
GPC6	Glypican-6	−9.3
HSPG2	Perlecan (heparan sulfate pg2)	−1.6
SRGN	Serglycin	52.9
BGN	Biglycan	−6.2
EPYC	Epiphycan	−8.9
LUM	Lumican	−16.5
DCN	Decorin	−6.8
PRG4	Proteoglycan 4	7.4
PRG2	Proteoglycan 2, bone marrow	2.0
CSPG4	Chondroitin-sulfate pg4	3.2
DAG1	Dystroglycan 1	−1.6
**HS BIOSYNTHETIC/MODIFYING ENZYMES**		
EXT1	Exostosin-1	−2.8
HS2ST1	Heparan sulfate 2-*O*-sulfotransferase-1	1.5
HS6ST1	Heparan sulfate-6-*O*-sulfotransferase-1	−3.5
NDST1	*N*-Deacetylase/*N*-sulfotransferase	−2.1
SULF-1	Sulfatase-1	−52.3
PAPSS1	3′-phosphoadenosine 5′-phosphosulfate synthase 1	−1.9
PAPSS2	3′-phosphoadenosine 5′-phosphosulfate synthase 2	1.7

*FC represents fold changes at *q* ≤ 0.05 of a gene following syndecan-1 overexpression compared to cells transfected with the corresponding vector control*.

**Figure 3:**
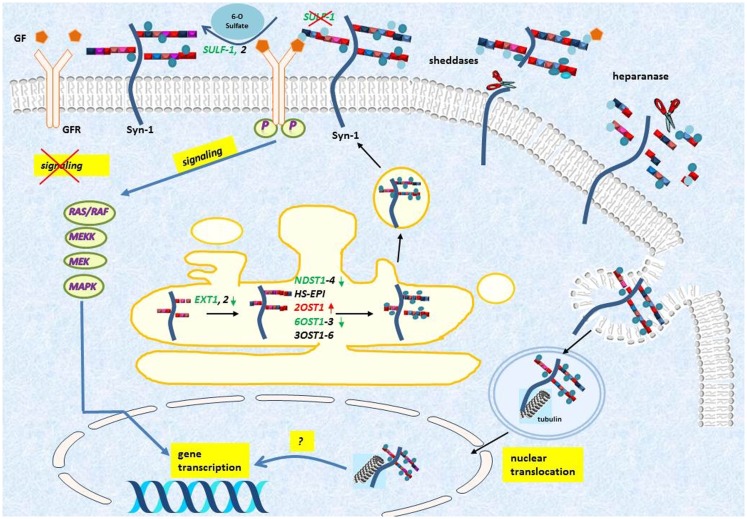
**Syndecan-1 turnover and its effect on HS modifications in malignant mesothelioma**. Syndecan-1 (Syn-1) is synthesized in Golgi and it is transported to cell-membrane where it acts as a co-receptor for various growth factors (GFs) and growth factor receptors (GFRs). The ectodomain is released from the cell-surface by the action of various enzymatic reactions collectively called sheddases, and the heparan sulfate can be further fragmented by the action of heparanases. The shedding results in a soluble molecule, which is still active and thereby can bind and sequester GFs. Syndecan-1 is also internalized and translocates to the nucleus in a tubulin dependent manner, but the function of this translocation is still incompletely understood. Upon syndecan-1 overexpression, several biosynthetic enzymes are modified including, EXT, exostosin; NDST, *N*-deacetylase/*N*-sulfotransferase; OST, *O*-sulfotransferase; HS-EPI, C5 epimerase; and they collectively lead to altered HS synthesis and sulfation pattern (Colors represent: red = upregulated and green = downregulated). The endosulfatase SULF-1, specifically removes the 6-*O*-sulfate groups from the HS chains, and thereby may inhibit growth factor signaling. Downregulation of SULF-1 by syndecan-1, detected at transcriptional level, may lead to modulation of downstream growth factor signaling.

The massive downregulation of SULF-1 after syndecan-1 overexpression in malignant mesothelioma may constitute one mechanism by which syndecan-1 regulates cell growth, by modulating the growth factor binding properties of HSPGs (123) (Figure 3). Similarly, SULF-1 has a dual role in enhancing or inhibiting various growth factor signaling pathways and by that tumor cell proliferation: as it has a tumor suppressor role in the majority of carcinomas. SULF-1 is downregulated in many tumor types (124, 125), whereas in malignant mesothelioma and some other tumors it is overexpressed (124, 126). The mechanism of this dual effect has been ascribed to inhibition of the activity of FGF (127–129), HB-EGF (130), ERK-MAP, and AKT signaling pathways (131, 132). At the same time SULF-1 is known to promote WNT signaling (133, 134) and activates BMP/Noggin signaling (135). Currently it is hypothesized that cancers driven by WNT-1 signaling would likely be stimulated by SULF-1, whereas tumors depending on FGF-2 or HGF signaling as the most significant driving mechanism are inhibited (125, 136). In malignant mesothelioma the level of SULF-1 is elevated compared to the normal mesothelium and the Wnt pathway is also altered (137–139), thus we can hypothesize that SULF-1 downregulation contributes to inhibition of proliferation, however, the functional significance of these findings necessitates further investigations.

Syndecan-1 not only regulates multiple levels of HS biosynthesis, but also coordinates the expression of various PGs and fine tunes their regulatory pathways. Experimental data suggest also a cooperation between the different members of the syndecan family (92). Syndecan-1 seems to control the expression of other HSPGs in mesenchymal tumors, although the effect varies in different cell-types and also in the same tumor with various differentiation. Overexpression of syndecan-1 resulted in a downregulation of syndecan-2 and upregulation of syndecan-4 in epithelioid mesothelioma cells (90, 99), whereas in epithelioid fibrosarcoma cell line syndecan-2 was upregulated (92), and in a sarcomatoid fibrosarcoma cell line syndecan-4 was downregulated (90).

This overexpression of syndecan-1 in malignant mesothelioma cells was also associated with considerable changes in expression of other HSPGs: glypican-3 was upregulated whereas glypican-6 and perlecan both were downregulated.

The fact that the expression of syndecan-1 can influence the whole proteoglycan pool is further supported by several independent studies, where a higher level of syndecan-1 is accompanied by perturbations in the proteoglycan profile and in the HS biosynthetic machinery. The cell-type specific nature of this process, however, has to be emphasized as shown also in breast cancer cells and glioblastoma (140, 141). Similar to malignant mesothelioma there seem to be a phenotype specific HSPG distribution in glioblastoma, the mesenchymal subgroup of glioblastomas typically having a worse prognosis (141).

Taken together, growth factor signaling seems to be delicately controlled by regulatory loops involving the syndecan-1 expression levels, its cellular localization and the sulfation pattern. Syndecan-1 itself regulates the expression of multiple PGs and coordinates the HS biosynthesis. Furthermore, modulation of syndecan-1 affects the biological behavior of mesenchymal tumor cells and this involves genes regulating cell growth, cell cycle progression, adhesion, migration, and extracellular matrix organization; orchestrated in a complex network of signaling pathways (99).

## Targeting Syndecan-1 in Cancer

Syndecan-1 offers a multitude of possibilities for novel therapeutic approaches and targeted therapies. Therapeutic options should, however, consider that the syndecan-1 expression differs significantly from one tumor type to another and its effect is highly divergent comprising both anti-proliferative and pro-tumorigenic effects. Thus, in tumors with elevated syndecan-1 level such as multiple myeloma or breast adenocarcinoma, applicable approaches comprise anti-syndecan-1 antibodies, knockdown of syndecan-1, competitive inhibitors or anti-angiogenic agents (142–145). Synstatin, a short peptide that mimics a sequence of syndecan-1 extracellular domain seems to be a promising anti-angiogenic agent (35).

In contrast, in tumors of mesenchymal origin, and generally in tumors where syndecan-1 is downregulated, other approaches should apply. One interesting concept concerns possible growth inhibition by using soluble HS oligosaccharides or overexpression of syndecan-1 in mesenchymal tumors to hamper crucial biological responses including proliferation and migration (12, 43, 90, 146–148).

Cell surface HSPGs are also promising for efficient intracellular delivery of macromolecules across biological membranes (149–157) and offer encouraging possibilities of developing novel targeted treatments (158–161). The design of intracellular drug delivery, however, requires an increased understanding of the physiological processes that mediate cellular communication and transport across the plasma membrane.

## Conflict of Interest Statement

The authors declare that the research was conducted in the absence of any commercial or financial relationships that could be construed as a potential conflict of interest.
